# Transnational patterns, social networks and self-help organizations for migrant women

**DOI:** 10.12688/openreseurope.17168.3

**Published:** 2025-03-20

**Authors:** Paolo Ruspini, Petko Hristov

**Affiliations:** 1 Education Science, Roma Tre University, Rome, Lazio, 00185, Italy; 2 Institute of Ethnology and Folklore Studies with Ethnographic Museum, Bulgarian Academy of Sciences, Sofia, 1000, Bulgaria

**Keywords:** migrant transnationalism, gender networks, self-help organizations, inclusion practices

## Abstract

Previous research shows the importance of building up self-help structures in a transnational perspective for the inclusion of migrant women who are fleeing their home countries because of war, violence, or different forms of vulnerability. The mobilization of self-help organizations through the intersection of transnationalism and gender is, in fact, a useful direction for a practice-oriented pedagogy directed both towards (1) the most vulnerable groups of women, or (2) those already empowered either as community leaders or network facilitators, other migrants and the whole native population. For this paper, we compare two video-interviews of refugee women collected in Bulgaria and Italy, which are important receiving countries either at the South-Eastern or Southern external border of the European Union. The research questions of this comparative assessment include: 1) How do refugee women organize themselves for mutual help? 2) How do they build their social networks through transnational practices to bridge with the local people? 3) How is the community or individual empowerment of refugee women achieved across ethnic lines or through gender? The final aim of this investigation is to analyse various patterns of social networks’ creation among refugee women originating from different socio-cultural contexts. The research findings might be useful to instil inclusion practices which are apt to refugee women empowerment.

## Introduction

This paper aims at comparing selected data collected in the framework of the COST Action “Women on the Move” (WEMov). The comparative assessment included two video interviews of refugee women collected in Bulgaria and Italy, which are important receiving countries either at the South-Eastern or Southern external border of the European Union.

Transnational migration, gender, and the creation of “self-help” structures originating from the social network theory are considered conceptual tools for the inclusion of a sample of refugee women. The investigation aims at analysing various patterns of social network creation among refugee women originating from different socio-cultural contexts. The research findings, although limited in their geographical and sample extent, might be useful to instil inclusion practices that are apt for refugee women empowerment.

Several study topics are pertinent to this investigation and merit discussion in this migratory comparison. Among these is the understanding that women who are refugees are dispersed throughout various social and geographic situations and do not form a monolithic group. In these situations, given the varying migration experiences of the participating women, local integration strategies may vary greatly. Inclusion and learning acquisition strategies still require social safety, especially for migrant women who are subject to various forms of exploitation.

The research questions of this comparative assessment include: 1) How do refugee women organize themselves for mutual help? 2) How do they build their social networks through transnational practices to bridge with the local people? (3) How is the community or individual empowerment of refugee women achieved across ethnic lines or through gender?

The research explores examples of self-organization and community mobilization through transnational and ethnic networks and through gender mobilization in the host countries of refugee women seeking asylum.

## Methods

The article moves from a review of relevant theoretical concepts to the described migration process, ethnographic considerations and the proposed setting up of self-help structures for refugee women. Subsequently, Italian and Bulgarian data are presented with a caveat pertaining to the limited characteristics of the selected sample for the video-interviews. The interviewed women for the visual project of the COST Action WEMov were selected by snowball sampling by experienced researchers because of their life stories as migrants residing in one of the Action’s member states. Considered participants were over 18 years of age and competent to agree to participate formally in the project. Demographic criteria that are relevant to participant selection include: age, family status, dependents, and educational level. Participants were asked to bring to a comfortable video interview setting an object that represents something important to them about their migration journey. Objects or prompts such as a photograph, heirloom, jewellery, or musical instrument could be used.

Considering that “vulnerability” may connote classification, self-classification, and the maintenance of a devaluing or inferior image for the real victim of, say, sexual assault or war trauma as in our research sample, all ethical considerations and precautions were taken before, during, and after the interview. Sensitive arrangements included the form of interpersonal communication, conversation in the preferred language of the participant and welcoming, comfortable setting. The way the research questions were presented was discursive, dialogic, and considerate to avoid creating new trauma or further “vulnerability” issues. In addition, the respondents showed a sincere dedication to the project's objectives and a willingness to share their personal narratives and experiences to help other women who are migrants or refugees facing comparable circumstances.

The interviews were carried out by the authors of this article according to the requirements and protocol of the COST Action WEMov, and were approved for online publication by the Action’s Chair in accordance with all the ethical norms for conducting scientific research of the European Commission and the provisions of the Declaration of Helsinki.

The authors have obtained written consent from the women interviewed for participation and for their faces and stories to be published and broadcasted for scientific purposes. Participants were given the opportunity to have their personal data anonymised with pseudonyms, as well as having specific personal information omitted if they desired. The interviewees were also advised that they could leave the study or rescind their contribution at any point in the research process. The relevant narrative and visual data collected were saved on a password protected computer. Edited sections of the video interviews are open access and can be watched on the WEMov website. ^1^

Data collection methods for the one-on-one interview included the following general themes and related prompts: the motivation for migration; the migrant journey; the life of the interviewee before and after migration; and her hopes for the future. The questions of the interview grid were flexibly adapted to the distinct categories of selected participants who were given the opportunity to check for them in advance and afterwards they were provided with the raw and edited video materials and transcripts for comments ^2^ ( WEMov Visual Project, 2022).

The WEMov research network protocol allowed the collection of only a few video interviews per country. This limitation has been addressed for this comparative article by considering complementary data on refugee women collected in Bulgaria during different time lapses, as well as previous research on migrant women in Italy originating from the Sub-Saharan region ( Lapov & Campani, 2017; Ruspini, 2019). Finally, interpretive and conclusive findings are discussed.

## State of the art

The literature review focuses on a few concepts and their interrelation, which are either significant in migration studies or relevant for the general aim of this article, as well as for answering the suggested research questions around the nature of the proposed self-help structures for the empowerment of women. These concepts include social networks, gender, ethnic mobilisation and migrant transnationalism.

### Social networks

According to Granovetter (1973), social networks are networks of relationships or ties between people that can differ in strength, kind, and length. Bourdieu (1986) asserts that social networks, posing as social and cultural capital, are potential resources that must be activated. These resources include assistance in getting employment and moral or financial support. Joining a group grants access to a network; nevertheless, a person's ability to access resources is contingent upon their standing within the relevant network.

Granovetter (1973) concentrates on the advantages and disadvantages of social connections. Strong ties to family members and close friends are unquestionable, but they frequently do not give the person access to significantly more resources. Weak links are those with people who are further distant from you, such as acquaintances; these relationships need more "work" to be activated, but they might give you access to quite different resources ( Granovetter, 1983). For this reason, weak links may be more advantageous than strong ties in terms of getting access to resources that are crucial to society.

Networks are crucial tools for migration since they offer pathways into a targeted nation or aid in integrating into a particular culture. Labour, personal (family), and so-called "illegal" migrant networks are the three main social networks that typically influence migration patterns. Each of these has costs and benefits for the participating migrants ( Boyd & Nowak, 2012). Furthermore, while networks are crucial for employment and social opportunities - especially for the most vulnerable - not all migrants rely on labour networks to obtain work, nor do they all rely on personal networks during the settling process. However, the goal of this paper is to investigate the real prerequisites for networking or bringing about changes in ethnic networks through the activation of bridging social capital.

Migrant networks are a long-standing feature of Italian experience. Since the start of immigration to Italy, associations have developed into helpful resources that offer networks of mutual support and solidarity, particularly in the 1970s. Organizations made up of migrant women often replicate the unique migration history of individual national groups (such as associations of women who were born in Cape Verde and the Horn of Africa in the 1980s), as well as the level of internal gender relations and group cohesiveness (mixed-gender and/or exclusively female associations). To support them in obtaining their rights and acquiring specialized skills to provide welfare services to the migrant population, a large number of migrant women are grouped on the basis of gender in multi-ethnic associations, many of which include Italian members ( Ruspini, 2019).

However, despite the fact that non-religious organisations and those affiliated with the Catholic and other churches have played a leading role in providing various forms of support and assistance to immigrants in Italy, other observations indicate that the vast majority of immigrants have not benefited from any support at all, frequently relying on their own resources and small networks and groups formed through local circumstances or migratory chains, as well as individual relationships formed with the natives. The “less fortunate” immigrants, on the other hand, who have fewer resources, have ended up slipping into the ranks of social exclusion ( Ruspini *et al.*, 2000).

Bulgaria’s migration experience during the decades of the socialist rule was too limited, like several other former socialist countries. In the decades before 1989, the labour migration of the Bulgarian population was constrained within the former Eastern Bloc, within the republics of the USSR, and from the 1970s also in some of the countries of Asia and Africa, such as Libya, Syria and Angola for example. After 1990, the country became part of the world migration system, and the flows of emigration and immigration abruptly changed their destinations. And while Bulgarians immigrate in masse to the West (regularly or irregularly), Bulgaria’s geographical position positively affects immigration flows from countries outside Europe, as it is one of the three countries that share a land bridge to Asia and the Middle East at the base of the Black Sea. Moreover, the admission of Bulgaria to the EU in 2007 and the following opening of the European labour market for Bulgarian citizens resulted in an increase in immigration flows from the former Yugoslav and Soviet republics, mainly of people of Bulgarian origin from the historical diaspora in these countries. The growing immigration also includes migrants from the Near and Middle East, Iran, Afghanistan and China ( Dzhengozova, 2009: 201), and in the last decade (after 2014) also from Ukraine and Armenia.

Since the 1990s, there have been active migration studies in the media on the image and perception of migrants and refugees, both in local communities and in Bulgarian society. The policies of the Bulgarian state regarding the adaptation of migrants and opportunities for socialization and inclusion in Bulgarian society are also discussed. From 2020, the Bulgarian Council for Refugees and Migrants at the UN Refugee Agency (UNHCR) regularly publishes the Academic Bulletin “Refugees: Today and Tomorrow”. On its pages, many Bulgarian researchers and experts discuss critical issues regarding the policies of the Bulgarian state and the EU in general for the reception and socialization of refugees, the difficulties they face in their adaptation, and stories of the refugees themselves. A special issue of the Academic Bulletin with the title “Forced migration and women” is dedicated to refugee women and their stories about forms of self-organization and ways of building social networks for mutual aid ( Refugees: Today & Tomorrow, 2021). Recently, concrete studies of the cultural adaptation strategies of immigrants in the EU’s poorest countries have also appeared ( Hristov *et al.*, 2023). Migrant associations, as well as those particularly composed of and/or involving refugee women, remain a topic for further research.

In summary, ethnic networking has been seen as positive for bringing people together, but it also carries the risk of confining an individual to “her/his” ethnic/national space, which can be dangerous for some people, especially women who have fled perilous situations in their home countries or have had unique migration experiences.

### Gender and ethnic mobilisation

This study also considers the role of ethnic networks and gender roles in social capital ( Bourdieu, 1986). Intercultural ties encourage the “bridging” of social capital, whereas intimate community relationships support the idea of “bonding” social capital. Building bridges across social capital enables people to collaborate and network across diverse backgrounds as well as inside heterogeneous groupings ( Putnam, 2000). Bridging social capital was also positively correlated with education, especially focusing on adults’ transversal competencies. On the one hand, education can be a powerful tool in the development of diversified social capital since it increases tolerance and social trust. On the other hand, informal and diversified components of social capital influence successful education ( Helliwell & Putnam, 2007; Putnam, 2000). This kind of social capital generates weak ties, as per Granovetter’s (1983) definition, but ties that are more outgroup-oriented are more likely to foster social inclusion.

At the associative level, ethnic mobilisation in the host society is a strategy that has the potential to garner a great deal of attention. The materialisation of two fundamental objectives: the preservation of ethnic identity and the political representation of the migrating communities, including membership interests like the advancement of equality of opportunities, civic engagement, citizenship rights, and the fight against discrimination are its main goals, according to Sardinha (2009).

### Migrant transnationalism

The transnational immigrant dimension is also relevant in this research endeavour since associations mobilise not only along ethnic lines in the host country but also across receiving-sending country contexts. In this sense, Faist’s (2000) idea of the “community without propinquity”, which links migrant social and symbolic ties to positions in networks and organizations in different geographical locations covering two or more nation-states, is deemed important. Similar social arrangements are made possible by time-space compression; hence, these links between contexts also offer a favourable environment for continuing rights and identity negotiation ( Mapril & Araújo, 2002).

The described mobilisation in a transnational social space as per Faist’s ( 1998, 2000) definition also represents a useful ground for the intersection of gender and rights, as well as a direction for selective mobilisation of the paper’s sample across different lines. In fact, migrant organizations bring transnational resources not only to the country of origin but also to the country of settlement ( Clarke, 2013). Their role and impact on the lives of individual migrants, rather than in one country or another, fits Faist’s transnational perspective and provides grounds for our research endeavour.

### Transnational migration, gender, and self-help structures

The potential for additional migration studies or for the empowerment of women by refugees may lie in the confluence of transnationalism and gender in mobilising self-help support strategies for migrant and refugee women. In this context, evaluating the value of migrants’ social and cultural labour as well as how women use specific social networks can be accomplished using a gender-sensitive approach to the study of migrant networks ( Salaff & Greve, 2004).

To capture the intersections between gender and class in ethnic enclaves, empirical research should consider the social class composition of the community in which the networks are operating. This research should be inspired by former theoretical output in this spatial context and focus on the gendered division of labour within receiving countries, which is a result of the segmented labour demand ( Boyd & Nowak, 2012). In this regard, a considerable body of research has been done on the degree to which migration has disrupted social ties. Prior studies have demonstrated the duty and challenge faced by migrating women in creating new networks of care within their host societies due to time restrictions resulting from the necessity to care for their children ( Purkayastha, 2005; Salaff & Greve, 2004). If migrant women leave their family behind in their native nations, transnational nurturing obligations complicate matters further ( Bernhard *et al*., 2009; Parrenas, 2005).

The mobilisation of self-help structures through the intersection of transnationalism and gender is therefore a direction for practice-oriented pedagogy directed towards the most vulnerable groups of women or those already empowered either as community leaders or network facilitators among the same migrant women, other migrants, and the native population as a whole ^3^. The network building needs to be adapted to the unique circumstances of the countries under investigation, i.e., Italy and Bulgaria, the different samples, their potentialities, and specific needs per country and local contexts. Therefore, we now examine the collected data in Italy and Bulgaria.

## The fieldwork in Italy and Bulgaria

### Italy

In the Italian case, the research sample includes life stories collected through video interviews carried out in the summer of 2022 of two young migrant women (28 and 33 years old) residing in Italy, one who migrated from Argentina (for love) and another from the Democratic Republic of Congo (for asylum), first to South Africa and then Italy ( Ruspini, 2022).

The identified themes for the purpose of the interview included life before and after migration, obstacles and challenges resulting from migration, inclusion processes, role of mediators, use of social networks, transnational practices, objects of migration brought across borders, and plans of the interviewees.

For this comparative assessment of refugee women between Italy and Bulgaria, we limit our analysis to the patterns of migration and networking of the Congolese M. ^4^, since she migrated for asylum.

M. left the Democratic Republic of Congo because of sexual violence, threats against her father, and violent assault against the whole family from the local army. Consequently, her family was separated, and she moved first with her sister to South Africa, where she received refugee status. Afterwards, because of local job insecurity and xenophobia, M. made a secondary movement since she fled to Italy, where, at the time of the interview, she was still under humanitarian protection and unemployed while studying the local language and taking care of her small child.

During the interview, M. declared that her family had been disrupted and uprooted because of forced migration. Consequently, her nostalgia for home and local food is manifested as follows:

*I miss my friends and mostly the food [from home], I mean you can’t get that kind of food, fresh food like that in any other place in the world than home... […]. The cassava leaves,* si *[yes]! That’s like my favourite! I like cassava leaves and it’s different, it’s just different, when you are just back home is different. It is totally different.*

As far as social networks are concerned, in M.’s case, relevant research issues include connection with family and friends left behind, assistance from local churches in the first country of settlement (South Africa) to get a visa for Italy, and any help in settling in the local contexts. At a later stage of the incorporation process, networking with local people (through babysitting, as in the case of the Argentinian participant) is deemed useful to extend connections.

Transnational practices, either through mobility or virtual ICT, are deemed important, particularly in connection with M.’s disrupted family. The interviewee has, in fact, a big sister in Canada but also a fiancée in Sweden, as per the following quote:

*Well, the joyful part of my life [now] is that I met my fiancée, I have a baby and meet some new people. […] He is from Nigeria. At the moment, he is in Sweden. That’s where we met actually, so he is there, currently working there, so yes, that’s how we met and got a baby together and got engaged this year*.

Life as a migrant woman in Italy brought M. relief from a history of sexual violence and family disruption in Congo, but also from the experience of xenophobia in South Africa. However, the difficulties of living in a new country as a refugee woman have been emphasized.

*[…] As a female refugee, it is not easy at all, you are here alone like in my case, I am here alone, I am alone in a new country and you do not know if you are going to come across bad people or not and thankfully enough for me, I came across good people and I was helped and that’s it, that’s the good part for me was meeting good people. So, the bad part, was learning the language, indeed […].*

At the time of the video-interview, the life of M. in Italy was also much permeated by networking for help, ties of solidarity and “sisterhood” to other African refugee women in the shelter where she used to live and where we did pick her up for the recording of the video-interview. Babysitting from other refugee women allowed her sufficient time for the second recording since the first attempt was disturbed by the unavoidable but joyful presence of her little two-year-old baby who was crawling, playing around, and making noise while we were chatting for the interview. Sharing of children’s toys, clothes, and food with other African refugees of different origins was part of M.’s daily experience in the shelter, as well as her reliance on the staff of the non-governmental organization Ballafon in Varese that ran the aid services as well as other charities in their local network. Learning the local language helped her to engage and adapt her social and cultural capital as acquired in the first country of asylum, i.e. South Africa to the specificities of the new asylum context, i.e. Italy. 

### Bulgaria

We now turn to the second case for our comparison by examining Bulgaria as a destination country for refugee flows. Historically, Bulgaria had already received waves of refugees, both ethnic Bulgarians after the Balkan Wars and after the First World War, as well as ethnic Armenians after the genocide in the Ottoman Empire (1915–1919) or ethnic Greeks after the Civil War (1946–1949). Some authors have counted four waves of political immigrants and refugees from Ukraine to Bulgaria before the current Russian military aggression ( Hristov, 2021: 45–54). However, the Russian-Ukrainian armed conflict in February 2022 led to an explosion of refugee waves to the neighbouring countries of Ukraine, including countries close to language and culture, such as Bulgaria. The conflict between the two countries arose in 2014, when pro-European Ukrainians' Euromaidan protests led to the replacement of the Ukrainian government of pro-Russian President Viktor Yanukovych. It turned into an armed conflict after the disputed referendum in Crimea, which was annexed by the Russian Federation, and demonstrations by pro-Russian groups in the Donbass in April 2014. What had been going on for several years as a local armed conflict between the armed forces of Ukraine and Russian separatists escalated on 24 February 2022 in Russia's armed aggression in the eastern regions of Ukraine.

Many of the representatives of the historical Bulgarian diaspora in the regions occupied by Russia chose the path to Bulgaria in search of freedom and asylum. Hundreds of thousands of refugees left their native places, running away from the war. In general, three major groups of displaced persons have been formed due to armed conflict: those considering leaving (2,217,717 people), refugees (3,077,398 people), and internally displaced people, which is the most representative group of 6,477,723 people, predominantly women and children ( UIDR1 (2022); see also Koroutchev, 2023).

In the Bulgarian case, the data published by the National Council of Ministers account for nearly 195,000 Ukrainian citizens who had crossed the borders of Bulgaria and over 91,000 who had chosen to stay within the country, of whom approximately 35,000 children. At the end of June 2022, nearly 120,000 people were registered for temporary protection ( BfU, 2022), while at the end of January 2023, this number increased to 151,700 ( UNHCR, 2023).

Most refugees seeking protection are of Bulgarian ethnic origin, since the largest Bulgarian historical diaspora has been formed in South-Eastern and Southern Ukraine, accounting for a population of more than 200,000 according to official data. Ukrainian refugees who arrived in Bulgaria came mainly from the same regions, that is Odessa district, and to a lesser extent Mykolaiv, Kyiv, Kherson, and Kharkiv ( BfU, 2022).

In general, Ukrainian refugees can legally stay and work in the country if they receive temporary protection, which has been granted automatically if they have been living in Ukraine before the 24 ^th^ of February 2022, and after obtaining a foreigner’s identity card ( BGfUA, 2022). All the interviewed Ukrainian refugees started to work in the tourist, service, and entertainment sectors and succeeded in adapting very quickly and learning the Bulgarian language at a relatively proficient level ( Hristov, 2021: 45–54).

As can be seen from the interview with a woman from the Berdyansk region published on the WEMov map ( Hristov, 2022), many of them also possess Bulgarian passports. Although the official power in Kiev does not recognize dual citizenship, local institutions show an understanding and tolerance for the population of Bulgarian origin. Most Bulgarians living in Ukraine have retained their Bulgarian language in archaic dialect form for 200 years. This helps them adapt easily after arriving at Bulgaria.

Thanks to a special decree of the Council of Ministers of Bulgaria enacted in 1993 (No. 103), young Bulgarians belonging to this historical diaspora can study at Bulgarian universities with state grants. As a result, thousands of young people from Ukraine have taken this opportunity over the years. After some time in Bulgaria, many of them were used to returning to their native places as teachers in Bulgarian language and culture. The Russian occupation interrupted this process in the occupied zones, as far as only the use of the Russian language is allowed there. However, good knowledge of the Bulgarian and Ukrainian languages makes many of these women successful facilitators for mutual support in several cities in the Bulgarian-Ukrainian regions, as is the case with our interviewees.

N. (52 years old) was born in Bogdanovka, which is in the region of Berdyansk, in South-Eastern Ukraine. Prior to the current war, she was a teacher at a local school. The occupation of her village by the Russian army took place a few days after the start of military aggression and caused her family to separate. Consequently, N.’s son ended up in Bulgaria, whereas her sister was in Zaporozhie in Ukraine, and her niece in Bogdanovka asked her documents to obtain a Bulgarian passport. N. was able to leave Ukraine thanks to the first open corridor for refugees from Melitopol and Mariupol, with the aim of seeking refuge in Bulgaria, where her son was settled. Escapes are long and unsafe. N. speaks of:

*We were rather afraid when going through the one (Russian) and the other (Ukrainian) checkpoints, but we had no problems. And finally, when we heard them speaking in Ukrainian, we realized that we were already in the free Ukrainian zone and calmed down. We travelled in a red cross truck, 23–24 people, including children. It was dangerous because you were going through mined areas. We covered a distance of 100 km in 5–6 hours, but thanks to God, everything went well*.

Within three days in Bulgaria, she received refugee protection and status that granted her safety. Six months later, she was able to organize the arrival in Bulgaria with her elderly parents and her sick husband. She found and met again the people who helped them to evacuate via Crimea (i.e., the Russian zone), Georgia, and Turkey to Sofia, for a total of five days. After that, she immediately began to work as a volunteer at the refugee support center and has been collaborating with her sister for some time at the Foundation “For Our Children” in Sofia. At the beginning of military aggression, a special programme was already established in this foundation for the support of Ukrainian children and their mothers, where N. is currently working as a facilitator. When asked if she liked working at this foundation, she answered:

*I really like it. We work on various projects with children and women from Ukraine. Working with Ukrainian children is close to me, I also worked with children in Bogdanovka. The children are different - not only Bulgarians, there are Ukrainians, there are Russian speakers also, etc. But I love working with them*.

N. plans are clear, as she wants to stay in Bulgaria near her son and family. When asked if she misses her home in Ukraine, she answers:

*Maybe a little yes, but I'm here next to my son. I always wanted to be close to him, and that's probably why I took the departure from Ukraine more calmly. Maybe if the circumstances hadn't turned out like this, I wouldn't have been in a hurry to leave Bogdanovka, but now I'm fine in Sofia*.

## A final comparative note

The exposed Bulgarian contribution proves the importance of ethnic networks across borders, particularly in conflict-ridden situations where family bonds are endangered and thus need to be re-created. This research finding seems in line with that of the video interview of Congolese refugees in Italy, although the geographical and historical circumstances are different.

Transnational practices are also vital in both life stories in helping refugees to maintain bonds with their family and relatives. Interestingly, the Bulgarian case shows the importance of providing help in the volunteer sector as a refugee woman facilitator for co-nationals. This evidence matches well with the article’s scope of investigating the setting up of self-help structures, where facilitators can assist newly arrived women in the context of migration and search for international protection.

While ethnic mobilisation was deemed useful for the satisfaction of basic needs in and around the shelter, travelling and networking with distant others coupled with the engagement of her growing social and cultural capital allowed M. to start a slow process of reconstruction of her life, meeting new people, including her fiancée, and fostering intercultural ties that are prominent for her future wherever in Italy or Sweden where her boyfriend lives. Similarly, N.’s life has been under a process of reconstruction in the current country of temporary protection, i.e. Bulgaria, where thanks to the closeness of her relatives, she owns the resources to bridge with and help others, while maintaining symbolic and virtual transnational ties with her close home country, that is, Ukraine. 

As time passes, long-term empowerment for both refugee women seems to be the expected outcome of the described processes of life rebuilding, thanks also to the self-help structures generated under the tragic circumstances of war or violence that they have experienced. Finally, networking with other refugee women and their children as natural coping strategies to their present situation, as well as their intercultural encounters with native people, might bring positive outcomes in terms of inclusive practices both for them and the whole local population.

## Data Availability

The data for the quoted interviews are part of the results of the COST Action CA19112 “Women on the Move” (WEMov) work and can be observed here: https://www.womenonthemove.eu/documentaries/ This online repository contains the following underlying data for citation: • Ruspini, Paolo (28 July 2022). *Interview of Monique Tschienda (from Democratic Republic of Congo to South Africa and then Italy).* Varese, Italy (length: 1:01mn). • Ruspini, Paolo (24 June 2022). *Interview of Cecilia Ferullo (from Argentina to Italy).* Varese, Italy (length: 2.10mn). • Petko Hristov (23 December 2022). *Interview of Nataliya Romaliyska (from Ukraine to Bulgaria).* Sofia, Bulgaria (length: 2.12mn). COST Action CA19112 implements data processing for the purpose of managing the image rights of the participants. The legal basis for this processing is the contract (Article 6.1 b.) of the General Data Protection Regulation (GDPR). The recipients of the data are the persons in charge of data processing within COST Action CA19112. The data collected is kept in a current archive at the grant holder, i.e. the University of Picardie Jules Verne (UPJV) for the entire duration of the COST ACTION CA19112 and then five years in an intermediate archive. Full (raw) data (i.e. not edited portions of the video interviews) are stored in the repository of the University of Picardie Jules Verne (UPJV) and can be accessed upon request. To exercise these rights, COST Action CA19112 can be contacted by e-mail: contact@womenonthemove.eu or by post: Université de Picardie Jules Verne – 1, chemin du Thil 80000 Amiens (France). The Data Protection Officer can be also contacted at: contact.cnil@u-picardie.fr Extended data including the interview grids “ WEMov Visual Project (2022), *Questions and guidelines*, 19.09.2022”, the forms “Women on the Move’s Visual Project – Consent form” and “Authorization for recording and broadcasting (Image/voice). Legal Age” of the study’s participants can also be accessed at the above repository of the University of Picardie Jules Verne (UPJV).

## Footnotes

^1^ https://www.womenonthemove.eu/documentaries/#interviews

^2^ Four major categories of migrants were identified for the fieldwork as follows: 1. Forced migrants: refugees and asylum seekers, transit migrants, people whose migration is related to gender persecution (including transgender migrants), people who have been subjected to human trafficking, environmental migrants; 2. Voluntary migrants: remigration through Europe (networking migration), returnees, expatriates, people taking part in chain migration, modern nomads, lifestyle migrants, internal to border migrants; 3. Second, third, fourth generation migrants (left-behind populations as a consequence of migration) and helpers; 4. Highly skilled category: elite, professionals from multinational companies and retired people, academics.

^3^ According to the website of the Interaction Institute for Social Change (2009), three categories of social change networks have been established for implementation and migrant empowerment with the aim of providing social support and change networking. They include: - The connectivity (emerged) network which connects people to allow easy flow of and access to information. - The alignment network which aligns people to develop and spread an identity and a collective value proposition. - The production (action) network which fosters joint actions for specific outcomes by aligned people.

^4^ Although the data has openly shared access in the WEMov website, we present the respondents only with the initials of their first names.
