# Supplementary material for: Transnational patterns, social networks and self-help organizations for migrant women
Source: Open Res Eur. 2025 Mar 20;4:67. Originally published 2024 Apr 12. [Version 3] doi: 10.12688/openreseurope.17168.3 (PMC11747294; doi:10.12688/openreseurope.17168.3)
Supplement: Peer review history

# Reviewer report: Reviewer response for version 3

**DOI:** 10.21956/openreseurope.21576.r52319

**Published:** 2025-03-27

**Authors:** Erka Caro

**Affiliations:** 1 University of Tirana, Tirana, Albania

Substantial improvements have been made in response to prior feedback. While some issues (especially theoretical application and analytical depth) are only partially resolved, the revised version shows clear effort to address core concerns, and the paper now holds stronger academic value.

 Recommendation: Accept with minor revisions

 (Focused on strengthening conclusion and deepening theoretical analysis in discussion)

If applicable, is the statistical analysis and its interpretation appropriate?

Not applicable

Is the study design appropriate and is the work technically sound?

No

Is the work clearly and accurately presented and does it engage with the current literature?

Partly

Are the conclusions drawn adequately supported by the results?

No

Are sufficient details of methods and analysis provided to allow replication by others?

No

Are all the source data and materials underlying the results available?

Partly

Reviewer Expertise:

NA

I confirm that I have read this submission and believe that I have an appropriate level of expertise to confirm that it is of an acceptable scientific standard.

# Reviewer report: Reviewer response for version 2

**DOI:** 10.21956/openreseurope.19924.r49942

**Published:** 2025-02-21

**Authors:** Irina Diana Madroane

**Affiliations:** 1 West University of Timisoara, Timișoara, Romania

The article contributes to the research area of transnational migration and gender, by examining comparatively how two women migrants in Italy and Bulgaria, a Congolese asylum seeker and a Ukrainian refugee, respectively, employ social capital and build social networks in the transnational space as a way of achieving (self-)empowerment. The women migrants’ use and development of self-help structures and social inclusion strategies are discussed through the lenses of ethnic mobilization and transnational networking practices, which shape and are shaped by the migrants’ actions. Methodologically, the authors opt for an in-depth study of video interviews with the two women migrants (life stories), carried out as part of the COST Action WEMov. The gender-sensitive, qualitative research approach zooms in on the historical and geographical specificities of transnational migration spaces, viewed as relevant for practice-oriented pedagogy and policymaking in support of women migrants. The research design is sound, and the study is coherent, with minor exceptions. Overall, it succeeds at meeting its objectives, bringing to the fore the benefits and limits of ethnic mobilization in juxtaposition with intercultural and transnational relations building, both articulated in relation to gender.

 I suggest a clearer phrasing of the third research question and a clarification of why ethnic mobilization is counterposed to gender in this question (I believe the authors mean “transnationalism and gender”, but the wording of the question could still generate some confusion). In the presentation of findings, ethnic mobilization in the host country is mostly counterposed to transnational networking between sending and receiving countries, so there is a question mark regarding the formulation of the research question. Further, while transnational networking practices are indeed correlated to gender in the article, gender also plays a role in ethnic networking, as mentioned in the text, so there is some lack of clarity there, as well. For clarity purposes, too, and if that is the case, it should be specified that “ethnic mobilization” is confined in the study to the host country (otherwise ethnic networks cut across borders and may be considered to have a transnational dimension, as the Bulgarian case actually reveals). Some of these aspects require further theoretical forays and, especially, a strengthening of the connections among them in relation to the empirical work. The difference between the two strategies seems to lie rather in the type of social capital they require, and the authors could make this more explicit in the theoretical presentation. It is also unclear how the discussion of “the intersections between gender and class in ethnic enclaves” in the theoretical review applies to the empirical study. If it is simply an example of transnational relations, that should be specified.  Further, while it apparent that “bridging” social capital plays an important role in the building of weak ties outside one’s ethnic group, the role of “bonding” social capital remains somewhat backgrounded in the analysis of findings.

If applicable, is the statistical analysis and its interpretation appropriate?

Not applicable

Is the study design appropriate and is the work technically sound?

Yes

Is the work clearly and accurately presented and does it engage with the current literature?

Partly

Are the conclusions drawn adequately supported by the results?

Yes

Are sufficient details of methods and analysis provided to allow replication by others?

Not applicable

Are all the source data and materials underlying the results available?

Yes

Reviewer Expertise:

Main areas: applied linguistics, discourse and rhetorical studies, public discourse analysis Secondary areas: media and communication studies, gender studies, transnational migration studies

I confirm that I have read this submission and believe that I have an appropriate level of expertise to confirm that it is of an acceptable scientific standard, however I have significant reservations, as outlined above.

# Response

**Published:** 2025-03-13

**Authors:** Paolo Ruspini

**Affiliations:** Education, Universita degli Studi Roma Tre, Rome, Lazio, Italy

We would like to thank you for your valuable insights. We have considered them all and re-arranged the third research question to make it clearer. We have also added one short underlying paragraph on the research objectives. The theoretical concepts addressed in the article have been also checked both in the text (see ethnic mobilisation) and the conclusive statements to make it sure that they are fully aligned to the presented empirical data. There are also other minor additions throughtout the text that we are confident make the article fully consistent. Finally, slight editing has been also accomplished.

# Reviewer report: Reviewer response for version 2

**DOI:** 10.21956/openreseurope.19924.r49648

**Published:** 2025-01-21

**Authors:** John Eade

**Affiliations:** 1 University of Roehampton, London, England, UK

Looking at the response to the feedback submitted by the reviewers including me it is clear that they have tried as hard as they can to improve the paper. So I do not think that we should hold up the paper from being indexed

If applicable, is the statistical analysis and its interpretation appropriate?

Not applicable

Is the study design appropriate and is the work technically sound?

Yes

Is the work clearly and accurately presented and does it engage with the current literature?

Partly

Are the conclusions drawn adequately supported by the results?

Partly

Are sufficient details of methods and analysis provided to allow replication by others?

Yes

Are all the source data and materials underlying the results available?

Yes

Reviewer Expertise:

global migration, cultural diversity, urban ethnicity, identity politics, community representation, pilgrimage

I confirm that I have read this submission and believe that I have an appropriate level of expertise to confirm that it is of an acceptable scientific standard.

# Reviewer report: Reviewer response for version 1

**DOI:** 10.21956/openreseurope.18554.r40111

**Published:** 2024-08-14

**Authors:** Nasreen Chowdhory

**Affiliations:** 1 Department of Political Science, Delhi University, Delhi, India

The article reads very well with a clear set of questions:

 1) How do refugee women organize themselves for mutual help? 2) How do they build their social networks through transnational practices to bridge local people? (3) Is community or individual empowerment of refugee women better for implementation through ethnic lines or a gender perspective?

 While the questions attempt to engage with the state of the art in a comparative framework, the article appears to have certain drawbacks:

1. The state the art literature is relevant and engaging especially network theories and gender as a category. However it would have been interesting if the authors had attempted to bring the empirical cases with that of the theoretical framework. 
2. The analysis seems rather disjointed 
3. A small section on methodology need to be added. 
4. Perhaps the authors can be encouraged to build on the case studies and the narratives and add a small section to re-engage with the theoretical framework, which then would lead to a coherent conclusion.   

If applicable, is the statistical analysis and its interpretation appropriate?

Not applicable

Is the study design appropriate and is the work technically sound?

Partly

Is the work clearly and accurately presented and does it engage with the current literature?

Partly

Are the conclusions drawn adequately supported by the results?

Partly

Are sufficient details of methods and analysis provided to allow replication by others?

Yes

Are all the source data and materials underlying the results available?

Yes

Reviewer Expertise:

Migration, citizenship and ethnonationalism

I confirm that I have read this submission and believe that I have an appropriate level of expertise to confirm that it is of an acceptable scientific standard, however I have significant reservations, as outlined above.

# Response

**Published:** 2025-03-13

**Authors:** Paolo Ruspini

**Affiliations:** Education, Universita degli Studi Roma Tre, Rome, Lazio, Italy

We wish to thank you for your very useful comments. While revising the second version of the article, they have been taken on board, particularly your re-alignnment request between the theoretical and empirical part of this work as well as that for a methodological section which has been added.Currently, we are unfortunately not in the position to support the arguments with further data/case studies. We have however slightly revised the third research question and fulfilled other minor revisions after the article passed peer-review and we had received a fourth review. Thank you, again!

# Reviewer report: Reviewer response for version 1

**DOI:** 10.21956/openreseurope.18554.r40108

**Published:** 2024-05-29

**Authors:** Erka Caro

**Affiliations:** 1 University of Tirana, Tirana, Albania

Overall, the paper presents an intriguing topic with the potential for engaging insights, yet falls short of delivering a comprehensive analysis. The introduction provides a broad overview but lacks specificity, with some content that could be better suited for footnotes. While the paper's premise holds promise, its execution disappoints upon closer examination.

 One glaring issue is the ambiguity surrounding the selection method, described as coincidental, which raises concerns about the study's rigor. Methodological limitations are evident but not acknowledged, contributing to the lack of clarity in the methodology section. Key details, such as the number of interviews conducted and whether data stratification was employed, are conspicuously absent, undermining the study's credibility.

 The revelation in the results section that only two video interviews were conducted is troubling, as this sample size is insufficient for drawing meaningful conclusions or facilitating comparisons. Additionally, the diverse backgrounds of the participants, hailing from Ukraine and the Republic of Congo, coupled with disparate motives for seeking asylum, further complicate any attempts at meaningful comparison.

 Furthermore, there are no  clear links between the literature and the results, particularly regarding the application of theoretical concepts like social networks, ethnic, and gender networks. The research questions posed at the outset remain largely unanswered, leaving the reader with a sense of incompleteness. The absence of a conclusive section compounds this issue, denying the opportunity for a synthesized interpretation of findings.

 Moreover, the decision to include fieldwork descriptions within the results section seems misplaced and could have been better integrated into the methodological session for a more coherent presentation.

 In summary, while the paper's subject matter holds promise, its execution is not up to expected standards. Addressing these shortcomings through improved methodological transparency, clearer theoretical application, and a more cohesive structure would significantly enhance the paper's scholarly value.

If applicable, is the statistical analysis and its interpretation appropriate?

Not applicable

Is the study design appropriate and is the work technically sound?

No

Is the work clearly and accurately presented and does it engage with the current literature?

Partly

Are the conclusions drawn adequately supported by the results?

No

Are sufficient details of methods and analysis provided to allow replication by others?

No

Are all the source data and materials underlying the results available?

Partly

Reviewer Expertise:

Migration, gender, labor issues

I confirm that I have read this submission and believe that I have an appropriate level of expertise to confirm that it is of an acceptable scientific standard, however I have significant reservations, as outlined above.

# Response

**Published:** 2024-06-03

**Authors:** Paolo Ruspini

**Affiliations:** Education, Universita degli Studi Roma Tre, Rome, Lazio, Italy

We will answer to this review by blocks of paragraphs starting from comments on (1) the paper's introduction:

- We do believe that the introduction is clear in terms of objectives and contents of the article. As far footnotes, the article went through an attentive review by ORE senior editors and indications came to include specific elements in the text.

On (2) "the ambiguity sorrounding the selection mehod":

- The research’s sample limitations have been properly acknowledged along the text and they overall pertain to the COST Action’s limitation when doing “research”. Snowball method has been used and “coincidence” pertains to a specific ORE request during review. Kindly check for our final answer to the other reviewer as well. Number of interviews conducted and related methods have been clearly exposed but, if necessary, we will point out once more that we were able to carry out two interviews per country.

On (3) "the video interviews":

- On the sample size: the matter has been already exposed. Let us add that the WEMov project requested two video interviews per member country. The drafted comparison has been based upon two refugees’ women who searched for help when fleeing and found support at different level by networking, thanks.

On (4) the "link between the literature and the results":

- There is a comparative section where preliminary findings are discussed. This is not a final, conclusive work just the start of possible analysis of the ongoing incorporation process.

As far (5) "fieldwork descriptions within the results section":

- This is indeed arguable since the comparative assessment allows the discussion of specific points not necessarily exposed earlier. We will keep this comment under consideration, anyway.

At last (6) on "methodological transparency":

- We spent several rounds of revisions to make the methods as transparent and ethical sound as possible. Thanks, anyway, for your comments.

# Response

**Published:** 2025-03-13

**Authors:** Paolo Ruspini

**Affiliations:** Education, Universita degli Studi Roma Tre, Rome, Lazio, Italy

We also wish to thank you for the above detailed comments and for allowing the article to pass peer-review. We have now uploaded for publication a third version of the article with minor theoretical revisions to address the arguments raised by the fourth reviewer. Thank you again!

# Reviewer report: Reviewer response for version 1

**DOI:** 10.21956/openreseurope.18554.r40103

**Published:** 2024-05-20

**Authors:** John Eade

**Affiliations:** 1 University of Roehampton, London, England, UK

This is a promising paper that tackles the highly relevant issue of developing self-help structures through transnational networks among women who have been forced to migrate. The paper tackles this issue by drawing on interviews with two women in different countries linked to three research questions.

 In my opinion, the paper's impact can be improved by the following:

1.  Consider the limitations of the social capital model as exposed in the wider literature. Putnam's approach has been particularly questioned and his perspective differs considerably from Bourdieu's so bringing them together with Granovetter raises important theoretical issues that should be addressed for the sake of clarity. The exploration by Bourdieu of the role played by cultural capital could be useful here, especially as migration requires people to learn the dominant 'rules of the game' in a new country.
2. The balance between the two case studies needs improving. There is more detail about the Bulgarian context and this weakens the comparison between Italy and Bulgaria and the conclusions drawn from this comparison. More information about the role played by self-help organizations in Italy would be very helpful since the person interviewed appears to have relied more on her personal transnational networks, especially her partner in Sweden with whom she shares a child. 
3. It would be helpful to know more about the relationship between these two women and other migrants (male as well as female) and 'the whole native population' since this plays a crucial role in learning the local 'rules of the game'. There are more indications about how this relationship works in the Bulgarian case study so again more balance is needed here.

In sum, then, a very interesting and promising paper that can be strengthened along the lines outlined above.

If applicable, is the statistical analysis and its interpretation appropriate?

Not applicable

Is the study design appropriate and is the work technically sound?

Yes

Is the work clearly and accurately presented and does it engage with the current literature?

Partly

Are the conclusions drawn adequately supported by the results?

Partly

Are sufficient details of methods and analysis provided to allow replication by others?

Yes

Are all the source data and materials underlying the results available?

Yes

Reviewer Expertise:

global migration, cultural diversity, urban ethnicity, identity politics, community representation, pilgrimage

I confirm that I have read this submission and believe that I have an appropriate level of expertise to state that I do not consider it to be of an acceptable scientific standard, for reasons outlined above.

# Response

**Published:** 2024-06-03

**Authors:** Paolo Ruspini

**Affiliations:** Education, Universita degli Studi Roma Tre, Rome, Lazio, Italy

We will answer to this review by the relevant blocks of paragraphs pertaining to improving  the paper's impact moving from (1) the "limitations of the social capital model":

- The triangulation of adopted theoretical concepts supported by relevant literature is deemed useful in developing self-help structures, particularly in conflict ridden situation as those briefly exposed as well as in bringing positive impact in self-leaning perspective. Cultural capital has been addressed under the guise of class and gender in ethnic networks and their positionality. Intercultural exposure as bridging factor can then bring much needed support considered the experienced traumatic situations.

On (2)  "improving the balance between the two case studies":

- Partnership brought far away emotional support, but concrete help came from the mentioned “sisterhood” in the local shelter. Unfortunately, we could not explore further the shelter and related refugees’ women dynamics for lack of time and resources.

As far (3) "the relationship between these two women and other migrants" is concerned: 

- This comment pertains to a different level of the inclusion process which the research could not address as specified in the final section of the current paper. It would be indeed good to track the interviewees now to see how they cope with life and draft on their incorporation process.

On the reviewer's final statement:

- Data limitation because of the specific characteristics of the COST Actions which do not allow and fund research has been clearly addressed along the article. Considering that and the overall positive assessment of this review, we do believe that the “not approved” status does not reflect properly the quality of the work we were able to carry out under limited conditions. Therefore, we do invite the reviewer to revise his final assessment accordingly, thanks!  
