# Supplementary material for: Transnational patterns, social networks and self-help organizations for migrant women
Source: Open Res Eur. 2025 Jan 10;4:67. Originally published 2024 Apr 12. [Version 2] doi: 10.12688/openreseurope.17168.2 (PMC11747294; doi:10.12688/openreseurope.17168.2)
Supplement: Peer review history

# Response

**Published:** 2024-06-03

**Authors:** Paolo Ruspini

**Affiliations:** Education, Universita degli Studi Roma Tre, Rome, Lazio, Italy

We will answer to this review by the relevant blocks of paragraphs pertaining to improving  the paper's impact moving from (1) the "limitations of the social capital model":

- The triangulation of adopted theoretical concepts supported by relevant literature is deemed useful in developing self-help structures, particularly in conflict ridden situation as those briefly exposed as well as in bringing positive impact in self-leaning perspective. Cultural capital has been addressed under the guise of class and gender in ethnic networks and their positionality. Intercultural exposure as bridging factor can then bring much needed support considered the experienced traumatic situations.

On (2)  "mproving the balance between the two case studies":

- Partnership brought far away emotional support, but concrete help came from the mentioned “sisterhood” in the local shelter. Unfortunately, we could not explore further the shelter and related refugees’ women dynamics for lack of time and resources.

As far (3) "the relationship between these two women and other migrants" is concerned: 

- This comment pertains to a different level of the inclusion process which the research could not address as specified in the final section of the current paper. It would be indeed good to track the interviewees now to see how they cope with life and draft on their incorporation process.

On the reviewer's final statement:

- Data limitation because of the specific characteristics of the COST Actions which do not allow and fund research has been clearly addressed along the article. Considering that and the overall positive assessment of this review, we do believe that the “not approved” status does not reflect properly the quality of the work we were able to carry out under limited conditions. Therefore, we do invite the reviewer to revise his final assessment accordingly, thanks!  
